# Machine learning classifiers for screening nonalcoholic fatty liver disease in general adults

**DOI:** 10.1038/s41598-023-30750-5

**Published:** 2023-03-03

**Authors:** Shenghua Qin, Xiaomin Hou, Yuan Wen, Chunqing Wang, Xiaxian Tan, Hao Tian, Qingqing Ao, Jingze Li, Shuyuan Chu

**Affiliations:** 1 Health Management Center, Guilin People’s Hospital, Guilin, China; 2 Philippine Christian University, Manila, Philippines; 3 Laboratory of Respiratory Disease, Affiliated Hospital of Guilin Medical University, Guilin, 541001 Guangxi China

**Keywords:** Non-alcoholic fatty liver disease, Epidemiology

## Abstract

Nonalcoholic fatty liver disease (NAFLD) is one of major causes of end-stage liver disease in the coming decades, but it shows few symptoms until it develops into cirrhosis. We aim to develop classification models with machine learning to screen NAFLD patients among general adults. This study included 14,439 adults who took health examination. We developed classification models to classify subjects with or without NAFLD using decision tree, random forest (RF), extreme gradient boosting (XGBoost) and support vector machine (SVM). The classifier with SVM was showed the best performance with the highest accuracy (0.801), positive predictive value (PPV) (0.795), F1 score (0.795), Kappa score (0.508) and area under the precision-recall curve (AUPRC) (0.712), and the second top of area under receiver operating characteristic curve (AUROC) (0.850). The second-best classifier was RF model, which was showed the highest AUROC (0.852) and the second top of accuracy (0.789), PPV (0.782), F1 score (0.782), Kappa score (0.478) and AUPRC (0.708). In conclusion, the classifier with SVM is the best one to screen NAFLD in general population based on the results from physical examination and blood testing, followed by the classifier with RF. Those classifiers have a potential to screen NAFLD in general population for physician and primary care doctors, which could benefit to NAFLD patients from early diagnosis.

## Introduction

Nonalcoholic fatty liver disease (NAFLD) is one of the most important causes of liver disease with approximately 25% in the estimated prevalence worldwide, and it will become one of major causes of end-stage liver disease in the coming decades^1,2^. NAFLD is also the most quickly growing cause of hepatocellular carcinoma in liver transplant in the United States^3^. However, NAFLD shows few or no symptoms until it develops into cirrhosis^4^. This process usually takes more than seven years^4^. Thus, NAFLD patients will benefit from early diagnosis and appropriate management.

Machine learning has been used to develop models to classify subjects with or without NAFLD^5,6^. However, those previous studies included subjects from the same center in 2010 or 2014. The health status of general people recently should be different from 7 or 12 years ago. Moreover, previous models used a plentiful of clinical features, which included results from physical examination, complete blood count, liver function testing, lipid panel, renal function testing and tumor markers^5,6^. Those complex features could help enhance the performance of models. However, they also reduce the simplicity and practicability when the model is applied by primary care doctors.

Therefore, in this study, we develop machine learning models to screen NAFLD patients from general population using data from annual health examination in 2021. We respectively adopted decision tree, random forest (RF), extreme gradient boosting (XGBoost) and support vector machine (SVM) algorithm to develop our models. All of those algorithms are classic in machine learning, and have stable performance in medical models^7^. They also could show important features in the model, which could provide clinical information on NAFLD screening. Moreover, our models included less candidate features than previous ones^5,6^. That could make more applicable for primary care doctors to screen NAFLD, and keep a good performance at the same time.

## Results

### Subjects’ characteristics

This study finally enrolled 14,439 subjects, including 4411 in NAFLD group and 10,028 in non-NAFLD group. The subjects in NAFLD group had higher body mass index (BMI) (NAFLD group vs non-NAFLD group = 27.2 ± 3.2 vs 23.5 ± 3.0), higher blood pressure (systolic blood pressure (SP): 132 ± 18 vs 122 ± 18 mmHg; diastolic blood pressure (DP): 78 ± 12 vs 71 ± 11 mmHg), higher red blood cell count (RBC) (5.22 ± 0.55 vs 4.95 ± 0.57 × 10^9^/L), higher white blood cell count (WBC) (7.32 ± 1.78 vs 6.50 ± 1.58 × 10^9^/L), higher monocyte count (MONO) (0.50 ± 0.16 vs 0.43 ± 0.15 × 10^9^/L), higher lymphocyte count (LY) (2.50 ± 0.76 vs 2.20 ± 0.62 × 10^9^/L), higher neutrophil count (NE) (4.10 ± 1.30 vs 3.66 ± 1.19 × 10^9^/L) and higher eosinophil count (EO) (0.19 ± 0.15 vs 0.17 ± 0.16 × 10^9^/L) than those in non-NAFLD group (Table 1).

**Table 1:** Subjects characteristics in NAFLD group and non-NAFLD group.

Variables	NAFLD group (n = 4411)	Non-NAFLD group (n = 10,028)	*P* values
Sex (male)	3377 (76.6%)	5394 (53.8%)	< 0.001
Age (year)	48.4 ± 13.4	45.9 ± 14.8	< 0.001
BMI (kg/m^2^)	27.2 ± 3.2	23.5 ± 3.0	< 0.001
Systolic blood pressure (mmHg)	132 ± 18	122 ± 18	< 0.001
Diastolic blood pressure (mmHg)	78 ± 12	71 ± 11	< 0.001
Complete blood count			
White blood cell count (10^9^/L)	7.32 ± 1.78	6.50 ± 1.58	< 0.001
Red blood cell count (10^9^/L)	5.22 ± 0.55	4.95 ± 0.57	< 0.001
Hemoglobin (g/L)	153 ± 14	144 ± 16	< 0.001
Hematocrit (%)	45.0 ± 3.7	42.8 ± 4.2	< 0.001
MCV (fL)	86.8 ± 7.5	87.0 ± 8.1	0.142
MCH (pg)	29.5 ± 2.8	29.3 ± 2.9	0.005
MCHC (g/L)	340 ± 10	337 ± 10	< 0.001
Platelet (10^9^/L)	263 ± 60	259 ± 59	< 0.001
RDW-SD (%)	42.8 ± 2.8	42.4 ± 3.0	< 0.001
RDW-CV (%)	13.7 ± 1.4	13.6 ± 1.5	< 0.001
PDW (%)	13.7 ± 1.9	13.7 ± 1.9	0.784
MPV (fL)	9.9 ± 0.9	9.8 ± 0.9	0.049
P-LCR (%)	24.2 ± 6.2	23.8 ± 6.3	< 0.001
PCT (%)	0.257 ± 0.053	0.252 ± 0.053	< 0.001
Neutrophil count (10^9^/L)	4.10 ± 1.30	3.66 ± 1.19	< 0.001
Lymphocyte count (10^9^/L)	2.50 ± 0.76	2.20 ± 0.62	< 0.001
Monocyte count (10^9^/L)	0.50 ± 0.16	0.43 ± 0.15	< 0.001
Eosinophil count (10^9^/L)	0.19 ± 0.15	0.17 ± 0.16	< 0.001
Basophil count (10^9^/L)	0.04 ± 0.02	0.04 ± 0.02	< 0.001
Neutrophil ratio (%)	55.5 ± 7.7	55.7 ± 8.0	0.131
Lymphocyte ratio (%)	34.5 ± 7.3	34.4 ± 7.5	0.376
Monocyte ratio (%)	6.8 ± 1.6	6.7 ± 1.7	0.004
Eosinophil ratio (%)	2.6 ± 1.9	2.6 ± 2.1	0.732
Basophil ratio (%)	0.5 ± 0.3	0.5 ± 0.3	0.817
Liver function			
TBIL (μmol/L)	11.4 ± 4.8	11.2 ± 5.2	0.013
DBIL (μmol/L)	4.44 ± 1.62	4.47 ± 1.72	0.259
IDBIL (μmol/L)	6.98 ± 3.36	6.72 ± 3.78	< 0.001
ALT (U/L)	33 ± 22	19 ± 15	< 0.001
AST (U/L)	25 ± 13	20 ± 13	< 0.001
Ratio of AST/ALT	0.91 ± 0.55	1.27 ± 0.60	< 0.001
γ-Glutamyltransferase (U/L)	55 ± 57	31 ± 34	< 0.001
ALP (U/L)	78 ± 20	71 ± 21	< 0.001
Total protein (g/L)	78.5 ± 4.1	77.7 ± 4.2	< 0.001
ALB (g/L)	47.4 ± 2.5	46.9 ± 2.6	< 0.001
GLO (g/L)	31.1 ± 3.9	30.8 ± 3.9	< 0.001
Ratio of ALB/GLO	1.55 ± 0.23	1.55 ± 0.23	0.689
Lipid panel			
Triglyceride (mmol/L)	2.71 ± 2.70	1.44 ± 1.13	< 0.001
Total cholesterol (mmol/L)	5.24 ± 1.02	4.89 ± 0.93	< 0.001
HDL-C (mmol/L)	1.18 ± 0.30	1.43 ± 0.34	< 0.001
LDL-C (mmol/L)	3.25 ± 0.79	2.83 ± 0.76	< 0.001
VLDL-C (mmol/L)	1.20 ± 1.20	0.64 ± 0.50	< 0.001

*BMI* body mass index; *MCV* mean corpuscular volume; *MCH* mean corpuscular hemoglobin; *MCHC* mean corpuscular hemoglobin concentration; *RDW-SD* standard deviation in red cell distribution width; *RDW-CV* coefficient variation of red cell volume distribution width; *PDW* platelet distribution width; *MPV* mean platelet volume; *P-LCR* platelet larger cell ratio; *PCT* thrombocytocrit; *TBIL* total bilirubin; *DBIL* direct bilirubin; *IDBIL* indirect bilirubin; *ALT* alanine aminotransferase; *AST* aspartate aminotransferase; *ALP* alkaline phosphatase; *ALB* albumin; *GLO* globulin; *HDL-C* high-density lipoprotein cholesterol; *LDL-C* low-density lipoprotein cholesterol; *VLDL-C* very low-density lipoprotein cholesterol.

Moreover, when compared with the subjects in non-NAFLD group, those in NAFLD group had an increased ratio of AST/ALT (0.91 ± 0.55 vs 1.27 ± 0.60), a higher level of alanine aminotransferase (ALT) (33 ± 22 vs 19 ± 15 U/L), aspartate aminotransferase (AST) (25 ± 13 vs 20 ± 13 U/L), γ-Glutamyltransferase (γ-GT) (55 ± 57 vs 31 ± 34 U/L), triglyceride (TG) (2.71 ± 2.70 vs 1.44 ± 1.13 mmol/L), total cholesterol (TC) (5.24 ± 1.02 vs 4.89 ± 0.93 mmol/L), low-density lipoprotein cholesterol (LDL) (3.25 ± 0.79 vs 2.83 ± 0.76 mmol/L) and very low-density lipoprotein cholesterol (VLDL) (1.20 ± 1.20 vs 0.64 ± 0.50 mmol/L), and a lower level of high-density lipoprotein cholesterol) (HDL) (1.18 ± 0.30 vs 1.43 ± 0.34 mmol/L) (Table 1).

### Model performance

**Figure 1:**
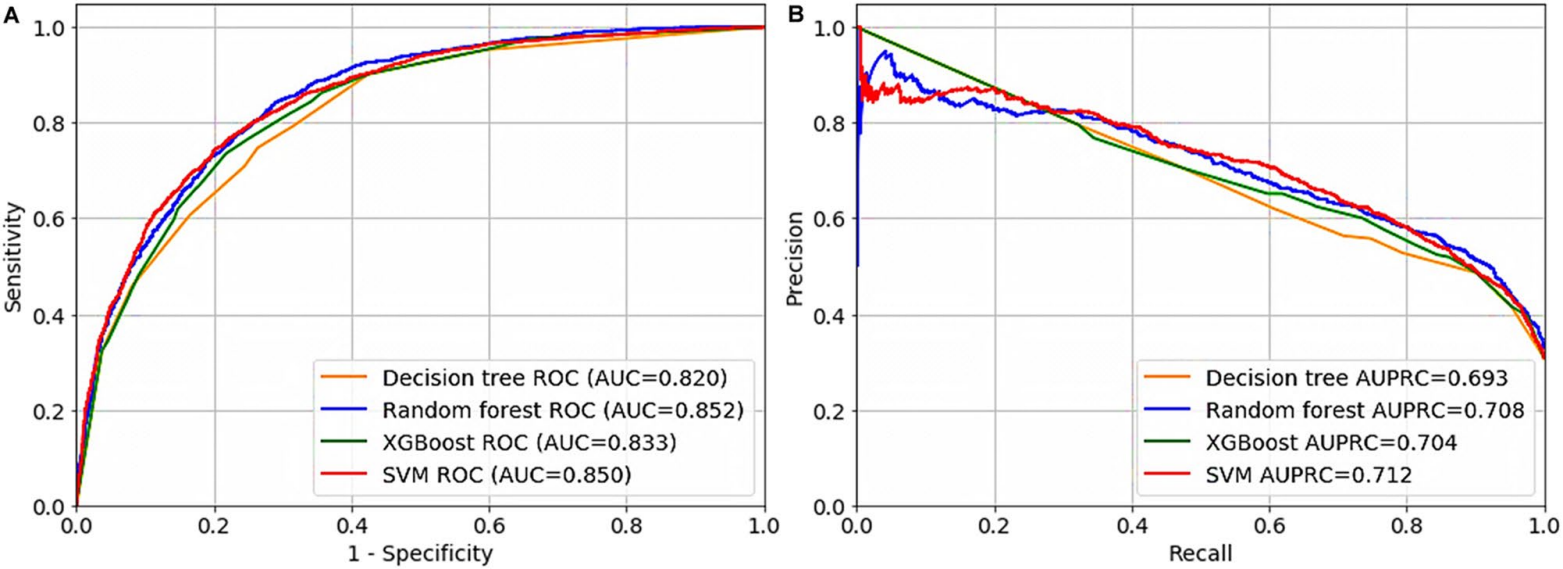
Receiver operator characteristic curves and precision-recall curve. (**A**) Receiver operating characteristic curve. (**B**) Precision-recall curve. *XGBoost* extreme gradient boosting. *SVM* support vector machine. *ROC* operating characteristic curve. *AUC* area under curve. *AUPRC* area under the precision-recall curve.

Table 2 and Fig. 1 illustrate the performance of all classifiers. The model based on SVM was showed the best performance with comprehensive evaluation, which included the highest accuracy (0.801), positive predictive value (PPV) (0.795), F1 score (0.795), Kappa score (0.508) and area under the precision-recall curve (AUPRC) (0.712), and the second top of area under receiver operating characteristic curve (AUROC) (0.850). The second-best classifier was that using RF, which was showed the highest AUROC (0.852) and the second top of accuracy (0.789), PPV (0.782), F1 score (0.782) and Kappa score (0.478) and AUPRC (0.708). The performance of XGBoost classifier follows RF (AUROC: 0.833, accuracy: 0.781, PPV: 0.778, F1 score: 0.779, Kappa score: 0.478 and AUPRC: 0.704). The worst performance was showed in the classifier based on decision tree.

**Table 2:** Model performance in testing dataset.

Model	Accuracy (95% CI)	PPV (95% CI)	F1 score (95% CI)	Kappa score (95% CI)	AUROC (95% CI)	AUPRC (95% CI)
Decision tree	0.765 (0.760–0.782)	0.763 (0.757–0.780)	0.764 (0.755–0.778)	0.444 (0.402–0.479)	0.820 (0.811–0.828)	0.693 (0.637–0.699)
Random forest	0.789 (0.781–0.796)	0.782 (0.774–0.789)	0.782 (0.775–0.790)	0.478 (0.458–0.498)	0.852 (0.843–0.852)	0.708 (0.694–0.715)
XGBoost	0.781 (0.767–0.786)	0.778 (0.758–0.780)	0.779 (0.757–0.781)	0.478 (0.411–0.482)	0.833 (0.818–0.837)	0.704 (0.623–0.704)
SVM	0.801 (0.789–0.801)	0.795 (0.781–0.795)	0.795 (0.781–0.795)	0.508 (0.475–0.509)	0.850 (0.840–0.850)	0.712 (0.694–0.712)

*PPV* positive predictive value; *CI* confidence interval; *AUROC* area under curve under the receiver operating characteristic curve; *AUPRC* area under the precision-recall curve; *XGBoost* extreme gradient boosting; *SVM* support vector machine.

### Important features from models

**Figure 2:**
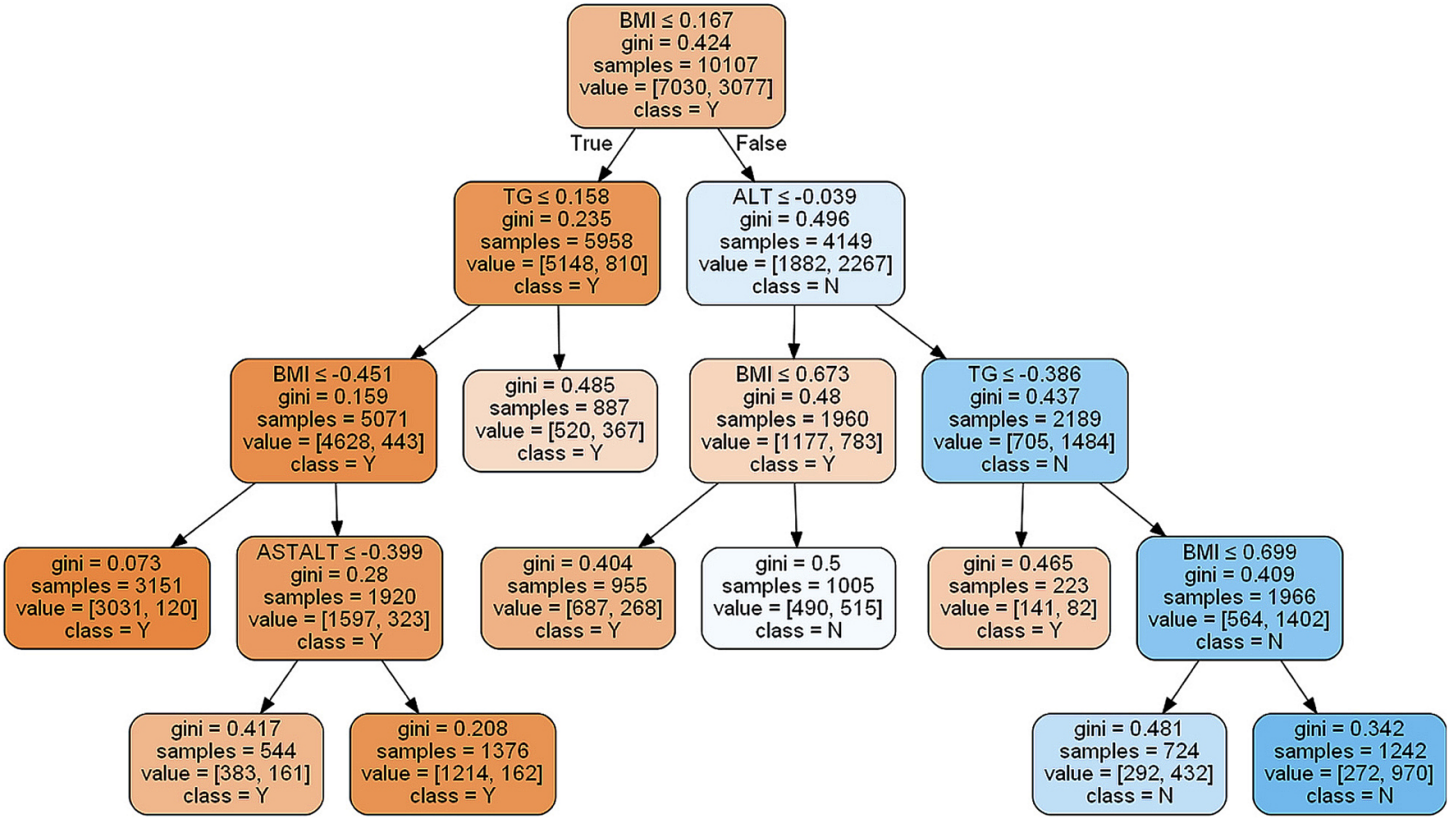
Classifier based on Decision tree. *BMI* body mass index; *TG* triglyceride; *ALT* alanine aminotransferase; *AST* aspartate aminotransferase; *ASTALT* the ratio of AST/ALT.

**Figure 3:**
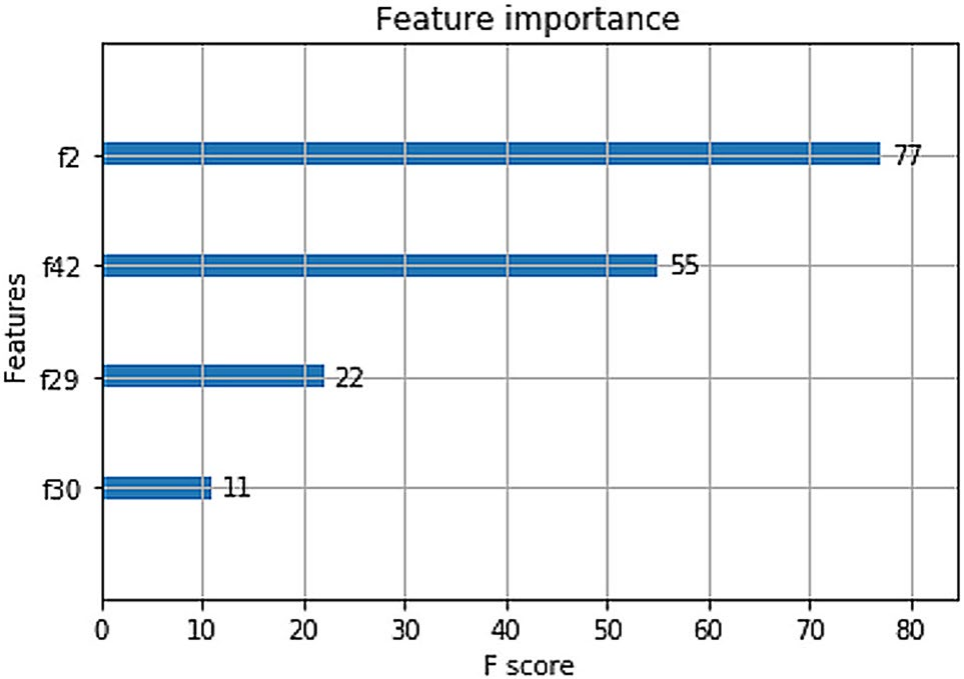
Features importance in classifier based on XGBoost. f2: BMI; f42: TG; f29: ALT; f30: ratio of AST/ALT. *BMI* body mass index; *TG* triglyceride; *ALT* alanine aminotransferase; *AST* aspartate aminotransferase.

All of the classifiers in the study could show important features in each model. For the classifier using decision tree, BMI, the level of TG and ALT, and the ratio of AST/ALT were the important features to decide the tree model (Fig. 2). The classifier from XGBoost showed the same features as that from decision tree (Fig. 3). The classifier based on RF also showed those exactly features and VLDL-C. For the classifier from SVM, more important features were showed in the model, including sex, age, BMI, hematocrit (HCT), mean corpuscular hemoglobin (MCH), mean corpuscular volume (MCV), mean platelet volume (MPV), platelet larger cell ratio (P-LCR), thrombocytocrit (PCT), platelet (PLT), hemoglobin (HGB), MONO, monocyte ratio (MONO%), eosinophil ratio (EO%), EO, ALT, AST, globulin (GLO), total protein (TP), LDL, TG, VLDL-C and TC.

## Discussions

In this study, we developed classification models using decision tree, RF, XGBoost and SVM to screen subjects with NAFLD from asymptomatic and general adults. All models were assessed by accuracy, PPV, F1 score, AUROC and AUPRC. The best performance was showed in the model based on SVM, followed by RF.

Our results showed that the model with SVM was the best one in performance, followed by RF model. RF model showed good performance in previous study on NAFLD screening^5^. SVM has been showed good performance in developing medical models, including those for classifying NAFLD patients^5,6,8^. Thus, our results were in consistent with previous studies^5,6^.

Moreover, our results showed that RF and XGBoost models performed better than decision tree, particularly the RF model. Since RF and XGBoost models integrated decision trees^9,10^, those models performed better than single decision tree model. In our study, the RF model was showed a bit better performance than XGBoost model. That may be partly related with our data, which were not absolutely balanced between the two groups (non-NAFLD group vs NAFLD group = 10,028 vs 4411). RF algorithm can balance the error of unbalance data and then get good result^9^. Even though, the performance of model from XGBoost was quite close to that from RF, and most of the assessed results for performance were within 0.01 between them (Table 2). Moreover, since our models based on the NAFLD prevalence in real world, our models may be more applicable than that from intentionally designed samples in the real world without an absolutely balanced sample.

The performance of our models in accuracy, PPV, AUROC and AUPRC was similar with those reported in previous studies^5,6^ (Supplementary table 1). When compared with models using the same algorithms in Liu’s work, the PPV in our models with XGBoost and SVM was similar with that in previous study^6^ (PPV: XGBoost: 0.778 vs 0.806, SVM: 0.792 vs 0.768). For AUROC, it’s was a bit better for XGBoost model in the previous one than ours, whereas it’s similar for SVM model in both studies (XGBoost: 0.833 vs 0.873; SVM: 0.850 vs 0.865). For AUPRC, models with XGBoost and SVM in Liu’s report was a bit better than ours^6^ (XGBoost: 0.704 vs 0.810; SVM: 0.712 vs 0.800). The accuracy in our models with RF and SVM was slightly lower than the previous one in Ma’s report^5^ (RF: 0.789 vs 0.827, SVM: 0.798 vs 0.827). In contrast, our models showed a bit higher F1 score than Ma’s report and Liu’s reported^5,6^ (Compared with Ma’s report: Decision tree: 0.764 vs 0.569; RF: 0.782 vs 0.579; SVM: 0.792 vs 0.557. Compared with Liu’s report: XGBoost: 0.779 vs 0.695; SVM: 0.792 vs 0.713). F1 score is a more appropriate index than accuracy score to assess the accuracy of model using data without absolute balance in both groups. Thus, our models were showed similar performances with previous one.

That previous study^6^ included features from physical examination, complete blood count, liver function testing, lipid panel, renal function testing and tumor markers. The more features, close to NAFLD or not, may increase the performance of models. However, more features and more complex of model could lead to more difficult in extending and application for primary care doctors. In contrast, the features in our models were simpler, which included results from physical examination, complete blood count, liver function testing and lipid panel (Supplementary table 2). Thus, our models may be more application in primary care practice, and also have a similar performance, when compared with the previous ones.

From our models, the most important features were BMI, the level of TG, VLDL-C, ALT and the ratio of AST/ALT in blood. Our results were in consistent with those in previous studies^5,6^. BMI and TG are risk factors for NAFLD in adults^11,12^, which were identified in all of our models. That may be related with an aberrant regulation of hepatic TG accumulation via de novo lipogenesis in NAFLD^13^. Moreover, the liver secretes TG in the form of VLDL for delivery to peripheral tissues. VLDL-C overproduction is one of characteristics of NAFLD^13^. That reflects an increased de novo lipogenesis plus lipolysis of intrahepatic and intra-abdominal fat in NAFLD^14^. In addition, in Chinese individuals without obesity, an increased ratio of ALT/AST is associated with the risk of new-onset NAFLD^15^. The decreased ratio of AST/ALT in NAFLD in our study was in consistent the previous finding^15^. That may be due to many reasons, for instance, a decreased AST/ALT ratio is related with chronic inflammation in liver, insulin resistance or steatosis of the liver, which leads to NAFLD^16,17^. Therefore, models in our study identified the importance of BMI, TG, VLDL-C, ALT and ratio of AST/ALT in NAFLD. And we further suggested those features should be important in screening NAFLD in general and asymptomatic adults.

We acknowledged that in our study, the diagnosis of NAFLD was based on abdominal ultrasound examination, instead of liver biopsy. That’s because liver biopsy was not permitted to screen NAFLD among general and health population according to ethic principles and the declaration of Helsinki. Abdominal ultrasound has been widely accepted to serve as an accurate and non-invasive tool in diagnosing NAFLD^18^. In our study, the prevalence of NAFLD was 30.5% (4411/14,439). That is similar with the national prevalence 29.2% in China, which was based on the database from 2008 to 2018 and has been increased over years^19^. Thus, the diagnosis of NAFLD from abdominal ultrasound could be accurate and reliable in our study. Moreover, type 2 diabetes mellitus (T2D) history wasn’t included into the candidate features. That’s because we can’t diagnose T2D from our subjects based on our data, and personal-reported history can’t be excluded bias. Since T2D is a risk factor of NAFLD^11,12^, the performance of our models should be improved if T2D was included as candidate feature.

In conclusion, the classification models could be developed using machine learning based on the data of annual health examination, which could screen NAFLD in general adults without redundant examination. The model using SVM is the best one, followed by that using RF. Those models may provide a readily available tool for physician and primary care doctors to screen NAFLD in general population, which could benefit to NAFLD patients from early diagnosis.

## Methods

### Study population

The subjects (age ≥ 18 years) were recruited into our study from general people who attended an annual health examination in Guilin People’s Hospital, from January to December in 2021. All laboratory testing and quality control were carried out by the laboratory analysis center of the hospital. The study protocol was approved by the Research Ethics Committee of Guilin People’s Hospital, and confirmed to the declaration of Helsinki. Written informed consent was obtained from each subject. NAFLD was diagnosed by exporters with at least 5 years’ experience, when there was evidence of hepatic steatosis by color Doppler ultrasound with a 3.5-MHZ probe (GE LOGIQ, Suzhou, China)^20^.

The subjects were excluded when they were: (1) had alcohol consumption > 210 g/week for men and 140 g/week for women; (2) had a history and/or laboratory evidences of viral hepatitis, autoimmune hepatitis, drug-induced liver disease or other chronic liver diseases, and secondary fatty liver; (3) in an acute or chronic infections; (4) in pregnancy or lactation; (5) had psychiatric disorders; or (6) had malignant tumor.

### Candidate features to classify subjects

The candidate features were collected from the electronic medical record, including age, sex, BMI (BMI = weight/height^2^), systolic blood pressure (SP), diastolic blood pressure (DP) and blood testing.

The blood testing was as following:(1) complete blood count: WBC, RBC, HGB, HCT, MCV, MCH, mean corpuscular hemoglobin concentration (MCHC), PLT, standard deviation in red cell distribution width (RDW-SD), coefficient variation of red cell volume distribution width (RDW-CV), platelet distribution width (PDW), MPV, P-LCR, PCT, NE, LY, MONO, EO, basophil count (BA), neutrophil ratio (NE%), lymphocyte ratio (LY%), MONO%, EO%, basophil ratio (BA%); (2) liver function: total bilirubin (TBIL), direct bilirubin (DBIL), indirect bilirubin (IDBIL), ALT, AST, ratio of AST/ALT, γ-GT, alkaline phosphatase (ALP), TP, albumin (ALB), GLO, ratio of ALB/GLO; (3) lipid panel: TG, TC, HDL, LDL, VLDL.

### Machine learning classifiers

The subjects were randomly divided into training set and testing set at a ratio of 7:3. The training set was used to develop the model, whereas the testing set was served to test the model. The data in training set were standardized using z-score transformation, and the data in testing set were transformed using the same parameters as those from the training data.

The models were developed based on training set by using Python3.7.6 programming language (http://www.python.org), scikit-learn22.2 library (https://scikit-learn.org/stable/). The models to classify subjects with or without NAFLD using decision tree, RF, XGBoost and SVM. SVM is an effective approach for classification by using linear functions or special nonlinear functions to transform the input space into a multidimensional space^21^. RF and XGBoost models respectively integrated decision trees based on a bagging combination of multiple tree models or a gradient increase framework^10,11^.

The hyper parameters with training set were estimated using grid-search and tenfold cross-validation. The best parameter combination in the model was selected when it led to the highest efficiency of the model. The gini criterion was selected with grid-search in the decision tree, which means CART tree was developed in our study. The performance of classifiers was tested on the testing set. The performance of models was assessed using accuracy, PPV, F1 score, AUROC, Kappa score and AUPRC.

### Statistical analysis

The Student’s t test or Mann–Whitney U test was used for comparing continuous data, and the Chi-square test was used for categorical variables. *P*-values < 0.05 were considered statistically significant. The statistical analyses were performed using SAS 9.4 (SAS Institute Inc., Cary, NC, USA).

## Supplementary Information

Supplementary Information.

## Data Availability

The dataset generated and/or analyzed during the current study are not publicly available but are available from the corresponding author and the first author on reasonable request. The code was based on Python3.7.6 programming language (http://www.python.org), scikit-learn22.2 library (https://scikit-learn.org/stable/). The codes are available from the corresponding author on reasonable request.
