# Supplementary material for: Machine learning classifiers for screening nonalcoholic fatty liver disease in general adults
Source: Sci Rep. 2023 Mar 3;13:3638. doi: 10.1038/s41598-023-30750-5 (PMC9984396; doi:10.1038/s41598-023-30750-5)
Supplement: Supplementary file 1 — Supplementary Information. [file 41598_2023_30750_MOESM1_ESM.docx]

Supplementary table 1. The performance of classifier in our study and the previous reports.

| Classifier | Studies | Accuracy | PPV | F1 score | AUROC | AUPRC |
| --- | --- | --- | --- | --- | --- | --- |
| Decision tree | Our study | 0.765 | 0.763 | 0.764 | 0.820 | 0.693 |
|  | Ma’s study [5] | 0.806 | / | 0.569 | / | / |
| Random forest | Our study | 0.789 | 0.782 | 0.782 | 0.852 | 0.708 |
|  | Ma’s study [5] | 0.827 | / | 0.579 | / | / |
| XGBoost | Our study | 0.781 | 0.778 | 0.779 | 0.833 | 0.704 |
|  | Liu’s study [6] | 0.795 | 0.806 | 0.695 | 0.873 | 0.810 |
| SVM | Our study | 0.801 | 0.795 | 0.795 | 0.850 | 0.712 |
|  | Ma’s study [5] | 0.827 | / | 0.557 | / | / |
|  | Liu’s study [6] | 0.798 | 0.758 | 0.713 | 0.865 | 0.800 |

PPV, positive predictive value; CI, confidence interval; AUROC, area under curve under the receiver operating characteristic curve; AUPRC, area under the precision-recall curve; XGBoost, extreme gradient boosting; SVM, support vector machine.

Supplementary table 2. Feature panel in the classifiers of our study and previous reports.

| Feature panel | Our study | Ma’s study [5] | Liu’s study [6] |
| --- | --- | --- | --- |
| Age | Yes | Yes | Yes |
| Sex | Yes | Yes | Yes |
| Physical examination results | Yes | Yes | Yes |
| Complete blood count | Yes | Yes | Yes |
| Liver function testing results | Yes | Yes | Yes |
| Lipid panel results | Yes | Yes | Yes |
| Renal function testing results | No | Yes | Yes |
| Tumor markers testing results | No | No | Yes |
